# Authentic Leadership Questionnaire applied to Brazilian nurses: evidence of validity

**DOI:** 10.1590/1518-8345.5868.3546

**Published:** 2022-07-15

**Authors:** Vanessa Gomes Maziero, Fernanda Ludmilla Rossi Rocha, Juliana Alvares Duarte Bonini Campos, Bruna Moreno Dias, Alexandre Pazetto Balsanelli, Carmen Silvia Gabriel, Andrea Bernardes

**Affiliations:** 1 Universidade de São Paulo, Escola de Enfermagem de Ribeirão Preto, Centro Colaborador da OPAS/OMS para o Desenvolvimento da Pesquisa em Enfermagem, Ribeirão Preto, SP, Brasil.; 2 Empresa Brasileira de Serviços Hospitalares, Campo Grande, MS, Brasil.; 3 Universidade Estadual Paulista, Faculdade de Ciências Farmacêuticas, Araraquara, SP, Brasil.; 4 Universidade Federal de São Paulo, Escola Paulista de Enfermagem, São Paulo, SP, Brasil.; 5 Bolsista do Conselho Nacional de Desenvolvimento Científico e Tecnológico (CNPq), Brasil.

**Keywords:** Leadership, Organization and Administration, Health Services, Nursing, Team, Validation Study, Psychometrics, Liderança, Organização e Administração, Serviços de Saúde, Equipe de Enfermagem, Estudo de Validação, Psicometria, Liderazgo, Organización y Administración, Servicios de Salud, Grupo de Enfermería, Estudio de Validación, Psicometría

## Abstract

**Objective:**

to establish the psychometric properties of the Authentic Leadership Questionnaire *(ALQ)* applied to Brazilian nurses.

**Method:**

cross-sectional observational study with a non-probabilistic sample. The psychometric properties of the RATER and SELF versions of the ALQ were calculated using confirmatory factor analysis with the WLSMV robust estimation method. The following indices were used to assess the goodness-of-fit of the model: chi-square by degrees of freedom (χ^2^/df), Tucker-Lewis Index (TLI), Comparative Fit Index (CFI), Root Mean Square Error of Approximation (RMSEA) and Standardized Root Mean Squared Residual (SRMR). Data reliability was analyzed using the ordinal coefficient alpha and composite reliability.

**Results:**

181 nurses participated of the study (female gender: 80.1%; mean age of 34.6 years; working time of less than five years: 76.3%). The complete ALQ RATER and ALQ SELF models did not present an adequate fit. Therefore, the refined models presented a better fit to the sample data (ALQ RATER: χ^2^/df=2.77; CFI=0.97; TLI=0.97; RMSEA=0.10; SRMR=0.05; ALQ SELF: χ^2^/df=2.74; CFI=0.94; TLI=0.92; RMSEA=0.10; SRMR=0.08). In the ALQ RATER model, items 1, 7 and 13 were excluded. Due to the high correlation between the factors Relational Transparency and Moral Perspective, a three-factor model based on the combination of the factors mentioned above was proposed. In the ALQ SELF model, items 2, 5, 9 and 10 were excluded. Likewise, a three-factor model based on the combination of two factors, now called Self-Awareness Balance, was proposed.

**Conclusion:**

the data obtained with the Authentic Leadership Questionnaire with Brazilian nurses were valid and reliable.

## Resumo

**Objetivo:**

estimar as propriedades psicométricas do *Authentic Leadership Questionnaire* (ALQ) aplicado a enfermeiros brasileiros.

**Método:**

estudo observacional transversal com amostragem não probabilística. As propriedades psicométricas das versões RATER e SELF do ALQ foram estimadas utilizando-se análise fatorial confirmatória com método de estimação robusto WLSMV. Como índices de qualidade de ajustamento dos modelos, foram utilizados: razão do qui-quadrado pelos graus de liberdade (χ^2^/gl), *Tucker-Lewis Index* (TLI), *Comparative Fit Index* (CFI), *Root Mean Square Error of Aproximation* (RMSEA) e *Standardized Root Mean Squared Residual* (SRMR). A confiabilidade dos dados foi analisada por meio do coeficiente alfa ordinal e da confiabilidade composta.

**Resultados:**

participaram 181 enfermeiros (sexo feminino: 80,1%; média de idade de 34,6 anos; tempo de trabalho inferior a cinco anos: 76,3%). Os modelos completos da ALQ RATER e ALQ SELF não apresentaram ajustamento adequado. Porquanto, o modelo refinado apresentou melhor ajustamento para a amostra (ALQ RATER: χ^2^/gl=2,77; CFI=0,97; TLI=0,97; RMSEA=0,10; SRMR=0,05; ALQ SELF: χ^2^/gl=2,74; CFI=0,94; TLI=0,92; RMSEA=0,10; SRMR=0,08). No modelo ALQ RATER, foram removidos os itens 1, 7 e 13. Devido à alta correlação entre os fatores Transparência Relacional e Perspectiva Moral, foi proposto um modelo trifatorial com base na junção dos fatores acima citados. No modelo ALQ SELF, foram retirados os itens 2, 5, 9 e 10. Do mesmo modo, foi proposto um modelo trifatorial baseado na junção de dois fatores agora denominado Equilíbrio Autoconsciência.

**Conclusão:**

os dados obtidos com *Authentic Leadership Questionnaire* com enfermeiros brasileiros foram válidos e confiáveis.

## Resumen

**Objetivo:**

estimar las propiedades psicométricas del *Authentic Leadership Questionnaire* (ALQ) aplicado a enfermeros brasileños.

**Método:**

estudio observacional transversal con muestreo no probabilístico. Las propiedades psicométricas de las versiones RATER y SELF del ALQ se estimaron mediante análisis factorial confirmatorio con el método de estimación robusta WLSMV. Como índices de calidad de ajuste de los modelos se utilizó: razón de chi-cuadrado por grados de libertad (χ^2^/gl), *Tucker-Lewis Index* (TLI), *Comparative Fit Index* (CFI), *Root Mean Square Error of Approximation* (RMSEA) y *Standardized Root Mean Squared Residual* (SRMR). La confiabilidad de los datos se analizó mediante el coeficiente alfa ordinal y la confiabilidad compuesta.

**Resultados:**

participaron 181 enfermeros (sexo femenino: 80,1%; edad media de 34,6 años; tiempo de trabajo inferior a cinco años: 76,3%). Los modelos completos ALQ RATER y ALQ SELF no mostraron un ajuste adecuado. Por lo tanto, el modelo refinado presentó un mejor ajuste para la muestra (ALQ RATER: χ^2^/gl=2,77; CFI=0,97; TLI=0,97; RMSEA=0,10; SRMR=0,05; ALQ SELF: χ^2^/gl=2,74; CFI=0,94, TLI=0,92, RMSEA=0,10, SRMR=0,08). En el modelo ALQ RATER se eliminaron los ítems 1, 7 y 13. Debido a la alta correlación entre los factores Transparencia Relacional y Perspectiva Moral, se propuso un modelo de tres factores basado en la unión de los factores mencionados anteriormente. En el modelo ALQ SELF se eliminaron los ítems 2, 5, 9 y 10. Asimismo, se propuso un modelo trifactorial basado en la unión de dos factores, ahora denominado Equilibrio de Autoconciencia.

**Conclusión:**

los datos obtenidos con el *Authentic Leadership Questionnaire* con enfermeros brasileños fueron válidos y confiables.

**Highlights**

(1) The validation of an authentic leadership questionnaire is innovative in the country.

(2) Authentic leadership is a model still little used in the Brazilian scenario.

(3) The validated scales will allow the assessment of nurses’ authentic leadership.

## Introduction

The concept of “authenticity” is related to positive psychology and has its roots in Greek philosophy. It has first been applied in the fields of sociology and education^1^, but, in 2003, the construct gained greater prominence in the area of management and leadership, and authentic leaders began to be defined as those who are aware of how they think and behave and are perceived by others as aware of their own values, moral perspectives and knowledge and of the strengths of others^1^.

Authenticity requires trust, optimism, resilience and high moral character and is related to being true to yourself. The theoretical model of Authentic Leadership is characterized by transparency in relationships, sharing of information and feelings, organizational commitment, and satisfaction in performance through conduct consistent with a system of personal values and convictions. Authentic Leadership can result in greater motivation to engage in leadership roles as it creates a healthy work environment and promotes leadership self-efficacy^2^. Furthermore, a study suggests that Authentic Leadership is associated with the quality of customer service^3^.

The American Association of Critical-Care Nurses considers authentic leadership as the main leadership model and claims that it is capable of creating and sustaining healthy work environments in critical care settings^4^. Based on the assumptions of this model, those who are led can find greater meaning in the activities performed and greater commitment to work, creating an environment that supports both leaders and their subordinates.

The theory that supports Authentic Leadership is based on four pillars: Self-awareness (leaders’ willingness to constantly analyze their strengths and opportunities for improvement); Relational Transparency (leaders’ ability to remain consistent with their values in the relationship with subordinates); Balanced Processing (unbiased decision making) and Internalized Moral Perspective (leaders’ values are consistent with their moral conduct)^5^.

Aiming to assess the authenticity of leaders, evaluating their ethical, moral, behavioral and organizational characteristics, in 2007, the original versions of the Authentic Leadership Questionnaire - ALQ^6^ were created and tested in two independent samples from the United States and China. The US sample consisted of employees of a high-tech industry, while the Chinese sample was composed of employees of a large state-owned enterprise. The ALQ was published in English in two different versions: the RATER scale, aimed at assessing the leadership exercised by the respondents’ leader; and the SELF scale, to assess the participant’s own leadership profile. Subsequently, validation studies of the ALQ were carried out for its use in various countries, such as Spain^7^, France^8^ and Pakistan^9^ ^-^ ^10^.

Criticism of the original 2008 study^6^ pointed out that the researchers did not report the use of modification indices related to the tests or the adjustment of models. In this sense, the authors encouraged other researchers to carry out new studies to explain and detail the analyzed performed^11^.

Recent research reported that the four factors that make up the ALQ (Self-awareness; Relational Transparency; Balanced Processing; and Moral Perspective) explain the composition of Authentic Leadership^11^.

It is important to emphasize that so far this instrument has not been validated in Portuguese nor for Brazilian people, and its psychometric properties have not been evaluated in a sample of nurses working in a hospital environment. However, as it one of the most used instruments to asses leadership style^10^, the cultural adaptation of the ALQ to Brazilian Portuguese and its evaluation by leaders working in the health area are urgent matters. This may help to design strategies that can contribute to a more effective leadership through fair and authentic management and transformative leadership.

Based on the above, this study was carried out with the objective of establishing the preliminary psychometric properties (validity of the internal structure) of the Authentic Leadership Questionnaire *(ALQ)* applied to Brazilian nurses.

## Method

### Study design

This is a cross-sectional observational study with a non-probabilistic sample, carried out in two general hospitals located in the Center-West Region of Brazil.

### Population and sample

The first institution (Hospital A) is a public teaching hospital with 210 inpatient beds in different specialties and approximately 1,600 professionals, of which 230 are nurses. The second institution (Hospital B) is a private, non-profit entity, under municipal management, with 671 beds and more than 3,600 professionals, of whom 260 are nurses^12^.

The target population of this study was composed of nurses working in these institutions. The inclusion criteria were nurses with an active bond with the institutions, and a total of 300 nurses were considered eligible. The minimum sample size for the factor analysis was calculated considering five to 10 subjects per parameter of the factor model^13^. As the ALQ (SELF and RATER) has 38 parameters (16 items, 16 errors and 6 correlations between factors), the minimum sample size estimated ranges between 190 and 380 participants. Nurses were approached individually at their workplace and invited to participate in the study. On that occasion, the two versions of the ALQ were delivered in printed form and then collected after five days. In this way, the participant was able to respond to the instrument with attention, at their own time and space, reducing the risk of interruptions and/or exposure and embarrassment. Of the 300 eligible nurses, 250 received the printed instruments and 50 were not located due to vacation, absence, relocation, among others. Of the 250 instruments delivered to nurses, only 200 were returned. Data was collected from December 2017 to March 2018.

### Instruments

A questionnaire composed of demographic and occupational questions was elaborated to characterize the sample. The questionnaire included: gender (female or male), degree (undergraduate, specialization/residence, master’s/doctorate), work sector (Administration/Management, Clinic/Surgical Unit, Maternal-Infant Care, Emergency Medical Care, Coronary Care Unit, Adult Inpatient Unit, Intensive Care Unit, among others), shift (morning, afternoon, evening, full-time), time of professional experience (in years) and time working in the hospital (in years).

The measurement instrument used was the Authentic Leadership Questionnaire - ALQ, which was applied to all participants in its two versions with different purposes: the RATER scale was used to assess the leadership of the respondents’ leader and the SELF scale was used to assess the leadership profile of the respondents’ themselves.

Each version (SELF and RATER) has 16 items distributed in four factors: Relational Transparency (RT - items 1 to 5); Moral Perspective (MP - items 6 to 9); Balanced Processing (BP - items 10 to 12); and Self-awareness (SA - items 13 to 16). The responses are on a five-point Likert scale (0: Rarely/never, 1: Occasionally, 2: Sometimes, 3: Often and 4: Very often, almost always)^6^.

The authorization to use the ALQ was granted directly by Mind Garden, Inc*.*, the company responsible for licensing the questionnaire, representing Dr. Bruce J. Avolio.

### Psychometric properties of the ALQ - evaluation of the internal structure

The psychometric sensitivity of the items, the factorial, convergent, and discriminant construct validity, and the reliability of the instrument for the sample (internal structure) were estimated.

Psychometric sensitivity was estimated using summary (mean, median and standard deviation) and shape measures (asymmetry and kurtosis) of the distribution of the responses to the items and was considered adequate when the absolute values of asymmetry and kurtosis were less than three and seven, respectively^14^ ^-^ ^15^.

The factorial validity was tested by means of Confirmatory Factor Analysis (CFA) using the robust estimation method of mean- and variance-adjusted weighted least squares (WLSMV). The following indices were used to assess the goodness-of-fit to the sample data: chi-square by degrees of freedom (χ^2^/df), Comparative Fit Index (CFI), Tucker-Lewis Index (TLI), Root Mean Square Error of Approximation (RMSEA) and Standardized Root Mean Squared Residual (SRMR). The following values were considered adequate: χ^2^/df ≤ 2.0; CFI and TLI ≥ 0.90; and RMSEA ≤ 0.10^16^ ^-^ ^17^. In addition, factor loadings (λ) were evaluated and considered adequate if ≥ 0.50. The modification indices calculated using the method of Lagrange multipliers (*LM*) were inspected to see if LM>11^13^ ^,^ ^15^.

Convergent validity was evaluated by Average Variance Extracted (AVE) and considered adequate if AVE ≥ 0.50^18^. The method used to assess the validity of the discriminant construct was the one was the one proposed in 1981^18^, which considered that if AVE_i_ and AVE_j_ ≥ ρ_ij_^2^, the existence of discriminant validity can be confirmed.

The reliability of the factors was evaluated using the ordinal coefficient alpha (α) and the Composite Reliability (CR). Values of α and CR greater than 0.70 were considered indicators of reliability^15^ ^,^ ^18^.

After the factor adjustment of the ALQ RATER and SELF scales, a second-order hierarchical model (SOHM) was proposed in order to verify the contribution of each factor to the general construct of leadership.

The programs MPLUS v.8.3 (Muthén and Muthén, 2019, Los Angeles) and R (R Core Team, 2016) with the packages “lavaan”^19^ and “semTools”^20^ were used to perform the analyses.

### Ethical aspects

The ethical precepts on the guidelines and standards for research involving human beings contained in Resolution No. 466 of the National Health Council of December 12, 2012 were followed. The study was approved by the Research Ethics Committee of the Nursing School of the University of São Paulo (CAAE: 67343717100005393).

## Results

Of the 300 eligible nurses, 200 responded to the questionnaire (adherence rate=66.7%) but 19 were excluded due to inconsistent responses, resulting in a sample of 181 participants. Among these, 63.5% (n=115) worked in hospital A and most were female (145; 80.1%). The mean age was 34.6 years old (standard deviation=6.7) and most participants had a specialization or residency degree (138; 76.2%), had worked in hospitals for less than five years (138; 76.2%) and had more than five years of professional experience (123; 67.3%). The demographic and occupational characteristics of nurses is presented in Table 1.

**Table 1:** Demographic and occupational characteristics of nurses (n=181). Campo Grande, MS, Brazil, 2018

Characteristic	No	%
**Gender**		
Female	145	80.1
Male	34	18.8
Did not answer	2	1.1
**Age group**		
20 to 29 years	47	26.0
30 to 39 years	92	50.8
40 to 49 years	37	20.4
50 years or older	3	1.7
Did not answer	2	1.1
**Highest degree**		
Undergraduate	21	11.6
Specialization/residency	138	76.2
Master’s/PhD	21	11.6
Did not answer	1	0.6
**Institution**		
Hospital A	115	63.5
Hospital B	66	36.5
**Work Sector**		
Intensive care unit	41	22.7
Other	41	22.7
Adult Inpatient Unit	29	16.0
Maternal-infant Care	23	12.7
Clinic/Surgical Unit	20	11.0
Coronary Care Unit	12	6.6
Emergency Medical Care	9	5.0
Administration/Management	6	3.3
**Work shift**		
Morning	58	32.0
Night	57	31.5
Afternoon	42	23.2
Full-time	23	12.7
Did not answer	1	0.6
**Professional experience**		
Less than 1 year	13	7.2
1 to 5 years	45	24.9
5 to 10 years	52	28.7
10 to 20 years	56	30.9
Over 20 years	14	7.7
Did not answer	1	0.6
**Time working in the Hospital**		
Less than 1 year	30	16.6
1 to 5 years	108	59.7
5 to 10 years	13	7.2
Over 10 years	30	16.6

**Figure 1:**
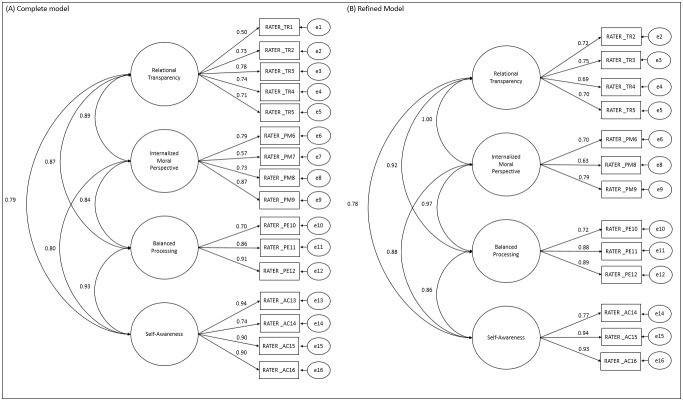
Confirmatory Factor Analysis of the complete (A) and refined (B) ALQ RATER models (n=181). Ribeirão Preto, SP, Brazil, 2021

Regarding the analysis of the psychometric properties of the ALQ for the sample, the CFA of the complete and refined models of the ALQ RATER are shown in Figure 1.

The complete model (A) of the ALQ RATER did not present an adequate fit to the sample data (χ^2^/df=4.42; CFI=0.94; TLI=0.92; RMSEA=0.14; SRMR=0.08). Items 1 and 7 showed low factor loadings and item 13 showed a high correlation with the instrument’s BP factor (LM=40.51-61.09); therefore, these items were removed. The refined model (B) showed the best fit to the sample data (χ^2^/df=2.78; CFI=0.98; TLI=0.97; RMSEA=0.10; SRMR=0.05). The convergent validity of the ALQ RATER scale factors was adequate (VEM=0.50-0.78). However, the discriminant validity of the instrument’s factors was not confirmed (r^2^=0.60-1.00) due to the strong correlations between them. As for reliability, acceptable values of ordinal α (0.74-0.91) and CR (0.69-0.88) were found.

**Figure 2:**
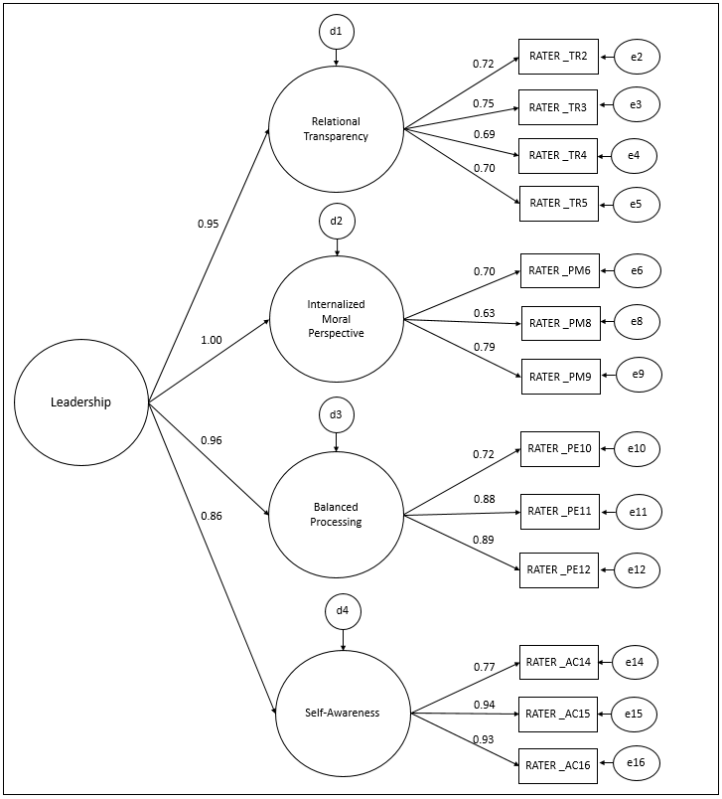
Confirmatory Factor Analysis of the ALQ RATER second-order hierarchical model (n=181). Ribeirão Preto, SP, Brazil, 2021

In order to assess the contribution of factors to the concept of leadership, a second-order hierarchical model (SOHM) was proposed for the ALQ RATER (Figure 2).

The SOHM of the ALQ RATER presented an adequate fit to the sample data (χ^2^/df=2.89; CFI=0.97; TLI=0.97; RMSEA=0.10), adequate factor loadings (λ=0.63-0.94) and high contribution of factors to the leadership construct (β=0.86-1.00; p<0.001).

**Figure 3:**
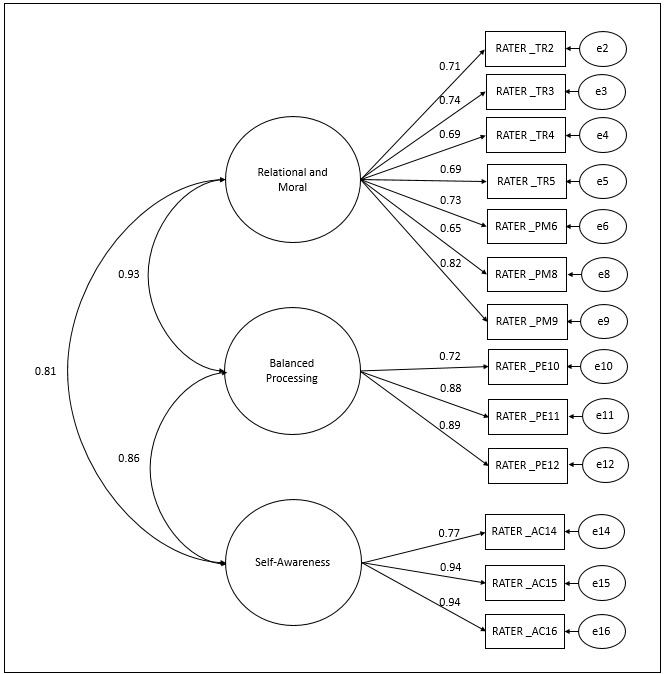
Confirmatory Factor Analysis of the ALQ RATER three-factor model (n=181). Ribeirão Preto, SP, Brazil, 2021

Nevertheless, based on the observation of the high correlation between the factors RT and MP factors (Figure 1), a three-factor model was proposed for the ALQ RATER (Figure 3), with the original factors BP and SA and a third factor combining the RT and MP factors (called Relational and Moral - RM), composed of items RT2, RT3, RT4, RT5, MP6, MP8, and MP9. The model presented an adequate fit to the sample data (χ^2^/df=2.77; CFI=0.97; TLI=0.97; RMSEA=0.10; SRMR=0.05).

This three-factor model presented factor loadings λ ≥0.65, strong correlations between factors (r=0.81-0.93), and adequate convergent validity (AVE=0.52-0.78). The discriminant validity between the factors was not confirmed (r^2^ =0.66-0.86) and adequate ordinal α (0.85-0.91) and CR (0.83-0.88) values were found.

As for the ALQ SELF, the complete four-factor model did not fit the sample data (χ^2^/df=3.85; CFI=0.84; TLI=0.81; RMSEA=0.13; SRMR=0.11). Most items had low factor loadings, and items 9 and 10 were correlated with other factors in the instrument (LM=48.15-61.22). There were moderate correlations between most factors (r=0.48-0.68) and strong correlations between the BP and SA factors (r=0.97).

**Figure 4:**
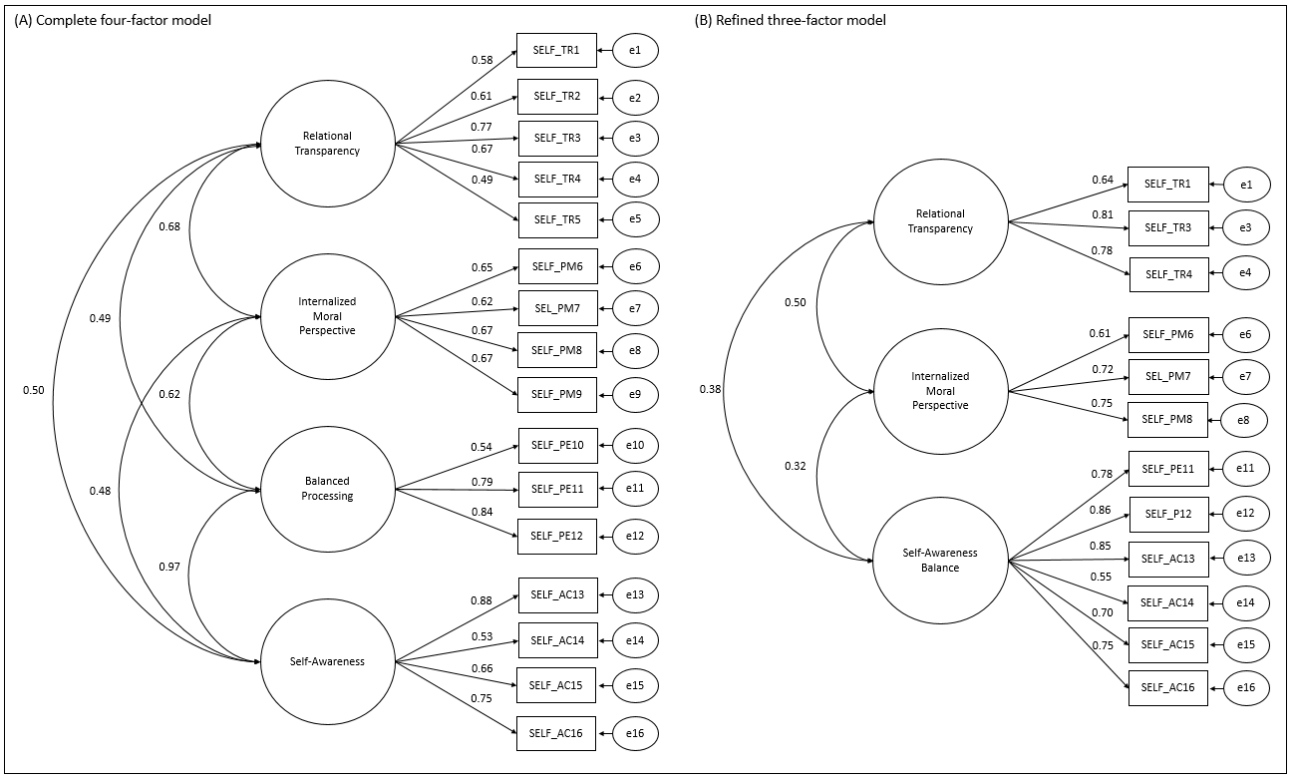
Confirmatory Factor Analysis of the complete four-factor (A) and refined three-factor (B) ALQ SELF models (n=181). Ribeirão Preto, SP, Brazil, 2021

However, during the adjustment, it was found that the BP factor would be composed only of items 11 and 12. Therefore, a three-factor model adjusted to the sample and combining the BP and SA factors was proposed. This three-factor model contained the factors RT, MP and the combination of BP and SA (called Self-Awareness Balance - SAB), composed of items BP11, BP12, SA13, SA14, SA15 and SA16. The model presented an adequate fit to the sample data (χ^2^/df=2.74; CFI=0.94; TLI=0.92; RMSEA=0.10; SRMR=0.08). The CFAs of the full model (four-factor) and of the refined model (three-factor) of the ALQ SELF are shown in Figure 4.

The convergent (AVE=0.48-0.57) and discriminant (r^2^=0.10-0.25) validity of the factors of the ALQ SELF refined were confirmed. Regarding the reliability of the items, adequate values were found (α=0.71-0.88; CR=0.67-0.84). Due to the moderate correlations between the factors, an ALQ SELF SOHM was not proposed for the sample.

## Discussion

In the ALQ RATER scale, the high correlation between the factors Relational Transparency and Moral Perspective made it possible to combine these factors. This is justified by the direct relationship between their theoretical concepts, since showing the true self to others (relational transparency) is an essential characteristic of those who behave according to their personal values (moral perspective). In addition, it is known that people tend to perceive and evaluate transparency based on their own structures, values, emotions and cognitive limitations^21^, and that relational transparency requires genuineness and transparency of leaders to gain the trust of their followers^22^, propagate their thoughts and seek harmony in the group^9^.

It is important to emphasize that it is necessary to invest in strengthening interpersonal relationships between leaders and team members, so that communication can be more assertive and self-confidence can be developed in the team^23^. Similarly, moral perspective requires a leader that can cultivate and promote values, so that they can make fair decisions based on high ethical standards^6^ ^,^ ^22^. Thus, the Relational and Moral factors are associated with transparency and ethics, which must go hand in hand. Corroborating the above, a study carried out in Belgium shows that the authentic leadership and behavioral integrity of the leader are related to follower performance and organizational commitment, and that this relationship is maintained by controlling the ethical organizational culture^24^, correlating the factors Relational Transparency and Moral Perspective.

The exclusion of items 1 and 7 of the new Relational and Moral factor and item 13 of the Self-Awareness factor is considered acceptable from a theoretical point of view, since the remaining items are capable of expressing the concepts of relational transparency (leaders show themselves as they are), moral perspective (leaders demonstrate values and ethical conduct) and self-awareness (leaders are aware of their strengths and weaknesses)^7^ ^,^ ^21^. Furthermore, the items are not always correlated with a single factor, as there is some degree of association with conceptually related factors^25^. In Spain, the ALQ was applied to a sample of 623 workers from public and private organizations from different sectors and, as in Brazil, there was a need to exclude three items^7^.

The second-order hierarchical model proposed for the ALQ RATER showed that the factors Relational Transparency, Moral Perspective and Balanced Processing were the ones that most contributed to the elaboration of the leadership construct with Brazilian nurses. This result differs from those of study carried out with 1861 employees from organizations of various sectors and sizes, which found that the dimension with the greatest explanatory power for the leader’s assessment was Self-awareness, followed by Balanced Processing^26^. In this sample, which compared 1019 Brazilian employees to 842 Portuguese employees, the theoretical explanation for this fact was that leaders’ self-knowledge and relationship with subordinates are essential for an authentic leadership^26^.

As in the RATER scale, the exclusion of items 2, 5, 9 and 10 can be theoretically justified, as it does not compromise the concepts of the different factors, since the remaining items can contemplate the definition of each one of the factors. It is also important to highlight that it is possible for the factors to overlap^25^, as occurred between Balanced Processing and Self-Awareness, which showed perfect correlation. For this reason, a single factor was proposed (Balance and Self-Awareness). This can be justified using the theoretical framework of the instrument itself, considering that in order to make a coherent decision it is necessary to analyze it beforehand (balanced processing) and to be aware of one’s own weaknesses and strengths (self-awareness). Balanced processing refers to the leaders’ ability to carefully analyze a situation before making decisions, while also being able to accept other points of view, even if they are different from their own^1^. Similarly, self-awareness refers to the deep perception of their values and beliefs and to how they behave and are perceived by others^1^ ^,^ ^27^. It is also worth noting that the creator of the instrument himself suggests that it is not reasonable to conceptualize the four factors of the ALQ as assessing entirely separate and distinct constructs^6^.

Given the above, the psychometric evaluation of the ALQ indicated the validity of the internal structure of the three-factor models of the RATER and SELF versions. In contrast, in Pakistan^9^, New Zealand^28^, Spain^7^, Belgium^24^ ^),^, Portugal^29^ and Turkey^30^, the validity of the four-factor model was confirmed^7^.

Regarding the differences in the adjustment of the two versions (SELF and RATER), when participants assess themselves as leaders, they are able to perceive balanced processing, being able, in their opinion, to make unbiased decisions; the same occurs with self-awareness, as individuals believe they understand the impact they can have on people^5^. However, the participants present difficulties in understanding the importance of relational transparency and moral perspective between the leader and the subordinates, which are characterized by high ethical standards guiding the behavior^5^ ^-^ ^6^.

In this investigation, the second-order hierarchical model was only possible for the RATER scale, which showed a strong correlation between the factors. This result corroborates the study by the creators of the ALQ^6^, which highlights that the four factors (Relational Transparency, Moral Perspective, Balanced Processing and Self-Awareness) are not independent and that the leadership construct, a single second-order factor, can explain this dependence.

The review of the validation by the authors of the original version of the ALQ^11^, in line with the original 2008 article^6^ reinforced the importance of the four theoretical components of the Authentic Leadership model. However, the authors encourage the development of other model validation studies^11^.

Thus, the proposed ALQ validation analysis may contribute to expanding the knowledge of professionals and researchers in the areas of nursing and hospital management, as the study presents the cultural adaptation of the ALQ to Brazilian Portuguese^31^ as well as evidence related to the validity of the instrument’s internal structure when applied to nurses working in Brazilian hospitals.

The limitations of this study refer to the use of a non-probabilistic sample and to the sample size, aspects that prevent the generalization of the results. However, it is worth noting that the SELF and RATER versions of the ALQ were considered valid and reliable instruments to be applied to the population of nurses, which will certainly contribute to the improvement of investigations on authentic leadership in Brazilian healthcare settings.

## Conclusion

The SELF and RATER scales of the Authentic Leadership Questionnaire (ALQ) applied to a sample of Brazilian nurses allowed the collection of valid and reliable information, which made it possible to assess the authentic leadership of these professionals. However, for the ALQ to be used, it was necessary to make adaptations in the instrument’s internal structure.

The SELF and RATER scales can be used independently and can be applied to Brazilian nurses working in hospital settings. The application of the scale may provide evidence related to the authenticity of the nurse leader and their influence in hospital organizations, opening space for reflection, discussion and future use of this tool for tracking and monitoring characteristics related to authentic leadership in this context. 
